# Transcriptome Analysis Unveils the Crucial Role of Mitochondrial Oxidative Phosphorylation Pathways in *Ulmus pumila* in Response to Salt Stress

**DOI:** 10.3390/plants15081164

**Published:** 2026-04-09

**Authors:** Yanqiu Zhao, Yu Guo, Shuo Song, Yongtao Li, Yuanyuan Shang, Zhaoyang Tian, Xiaoyu Li, Yihao Ding, Kaina Su, Chaoxia Lu, Dong Li, Lizi Zhao, Hongxia Zhang, Qingshan Yang

**Affiliations:** 1 Shandong Academy of Forestry, 42 Wenhua East Road, Jinan 250014, China; yanqiusunny@163.com (Y.Z.); liyongtao100@163.com (Y.L.); 2 The Engineering Research Institute of Agriculture and Forestry, Ludong University, 186 Hongqizhong Road, Yantai 264025, China; 15637057983@163.com (Y.G.); ss17658132181@163.com (S.S.); 18905433571@163.com (Y.S.); 15965943981@163.com (Z.T.); 15853470708@163.com (X.L.); dyh1314159@126.com (Y.D.); 18264483345@163.com (K.S.); ld@nwafu.edu.cn (D.L.); zhaolizi@126.com (L.Z.); 3 School of Biological Science and Technology, University of Jinan, 336 Nanxinzhuangxi Road, Jinan 250024, China; bio_luzx@ujn.edu.cn; 4 College of Agriculture and Forestry Science, Linyi University, Middle Section of Shuangling Road, Linyi 276000, China

**Keywords:** *Ulmus pumila*, salt stress, pathways, OXPHOS pathway

## Abstract

Elm (*Ulmus pumila*), an ecologically and economically valuable tree, exhibits significant tolerance to abiotic stress. However, the physiological and molecular mechanisms underlying its stress adaptabilities are largely unknown. Here, two elm salt-tolerant cultivars (ST-Y and ST-Q) and two salt-sensitive cultivars (SS-J and SS-JX) were identified in the 13 elm accessions collected from Shandong province, China via phenotypic salt tolerance screening. The key salt tolerance mechanisms were explored in ST-Y and SS-J via transcriptomic (RNA-Seq) assays, and subsequently validated in ST-Q and SS-JX via quantitative real-time polymerase chain reaction (RT-qPCR) analyses. Under salt treatment, ST-Y maintained leaf intactness and enhanced activation of antioxidant enzymes with a reduction in reactive oxygen species (ROS) accumulation, while SS-J suffered leaf defoliation and showed compromised antioxidant capacity with higher ROS levels. KEGG pathway analysis revealed that ST-Y leaves exhibited a unique enrichment of differentially expressed genes (DEGs) in the “oxidative phosphorylation (OXPHOS)” pathway after salt stress treatment. Both ST-Y and SS-J exhibited significant enrichment in the “metabolic pathway”, but the number of DEGs in the “arachidonic acid (AA) metabolism” pathway was much higher in ST-Y than in SS-J. Further RT-qPCR analysis verified the accuracy of the RNA-Seq data and revealed that genes related to the “OXPHOS” pathway were significantly up-regulated in ST-Y and ST-Q, but down-regulated in SS-J and SS-JX. Our results suggested that OXPHOS efficiency is critical to antioxidant capacity in elm salt tolerance, suggesting new avenues for forest tree improvement for climate change.

## 1. Introduction

Soil salinization impairs agroecosystems by inhibiting plant growth and reducing crop productivity, resulting in a range of negative effects on food security, ecological balance and long-term social and economic stability [1,2]. By 2050, an estimated 50% of the world’s fertile land could be affected by soil salinization, which would severely threaten the sustainability of agriculture and forestry [3,4]. Consequently, exploiting salt-tolerant plants and related genetic resources is essential for the ecological restoration of salinized soils and the development of sustainable forestry production.

Elm (*Ulmus pumila*), as a native species, is found in many places in northern China, and has important economic and ecological roles [5]. Elms provide multiple economic benefits: the high-quality wood of elm is prized for furniture production; elm fruits can be used as food ingredients; and the bark, roots, leaves and fruits have medicinal properties [6]. Beyond its economic significance, elm plants also have significant stress resistance [7]. The Yellow River Delta in Shandong Province harbors vast stretches of saline-alkali land, which presents significant obstacles to large-scale afforestation, and high-salinity conditions lead to low seedling survival rates and stunted growth, thereby severely hindering the development of the local forestry industry [8]. Therefore, the identification of salt-tolerant elm germplasm resources adapted to the local environment was essential for enhancing the efficiency of saline-alkali land restoration. Previous studies have shown that there are significant differences in the drought tolerance of elm trees due to genetic variation [9]. However, research on the salt tolerance of elm trees remains limited, with molecular mechanisms poorly understood, and the knowledge gap critically impedes the development of salt-tolerant cultivars through breeding and genetic improvement.

Plant salt tolerance involves complex regulation, integrating physiological, biochemical, and molecular responses to maintain cellular homeostasis under saline stress [10]. Under salt stress, plants activate coordinated physiological and biochemical responses to survive high-salinity conditions, including ion homeostasis, osmotic adjustment, antioxidant defense, and gene expression regulation [11]. A major challenge was the excessive accumulation of reactive oxygen species (ROS), which induced oxidative damage through membrane lipid peroxidation (e.g., elevated malondialdehyde (MDA) levels) and impairment of photosynthetic components (particularly photosystem II), ultimately causing cell death [12]. To counteract this, plants have evolved complex antioxidant systems involving enzymes such as superoxide dismutase (SOD), catalase (CAT), and ascorbate peroxidase (APX) that scavenge ROS and enhance stress tolerance [13]. Recent studies have shown that different species further optimize ROS homeostasis through unique gene regulatory networks. For instance, the SpsMDP1-WRKY53-PP2C80 module in willow (*Salicaceae*) activated the antioxidant enzyme system, reducing salt-induced oxidative damage [14]. Moreover, tomato (*Solanum lycopersicum*) exerted a non-enzymatic ROS scavenging function through the accumulation of branched-chain amino acids mediated by the SlAAP6 transporter protein, alleviating root stress damage [15]; IAA17.1-HSFA5a module regulated flavonol biosynthesis and controls ROS accumulation, thereby regulating the root system of poplar in response to salt stress [16]. Therefore, the above mechanisms provided important reference points for identifying salt tolerance pathways in elm.

In addition to oxidative stress responses, the energy metabolism homeostasis of plants plays a pivotal role in salt tolerance [17]. As the primary ATP source, OXPHOS requires tight coordination with antioxidant systems, with its efficiency directly determining cellular energy [18]. The OXPHOS-ROS relationship was particularly crucial, as electron transport chain (ETC) electron leakage serves as the major mitochondrial superoxide (O_2_^−^) source [19]. Studies have shown that optimizing the “OXPHOS” pathway could effectively reduce electron leakage and O_2_^−^ production [20]. Therefore, proper OXPHOS function was essential for ROS homeostasis [21]. As demonstrated in the halophyte *Suaeda salsa*, salt-induced upregulation of mitochondrial complex genes alleviates oxidative damage [22,23]. Maintaining precise levels of ROS was essential for cellular homeostasis [24]. Within this network, the arachidonic acid (AA) metabolic pathway was a critical signaling hub [25]. AA directly induces disease resistance in tomatoes and fine-tunes defense responses by interacting with other signaling molecules, such as jasmonic acid (JA) and salicylic acid (SA) [25,26]. Interestingly, overexpressing AA or its analogs in Arabidopsis exhibited enhanced resistance to biotic stress, yet displayed increased susceptibility to bacterial infection, revealing a dual role for AA in plant immunity [25]. However, no reports have been published on similar salt-tolerant metabolic networks in woody plants.

In this study, we compared the physiological responses of salt-tolerant and salt-sensitive elm cultivars to salt stress, focusing on the accumulation of ROS, the activities of antioxidant enzymes and the pathway response to salt stress. Subsequently, we identified the synergistic effects of the “OXPHOS” pathway and antioxidant enzymes in salt-tolerant elm cultivars in response to salt stress through RNA-Seq assay. This research will provide valuable candidate genes and a strong theoretical foundation for breeding salt-tolerant elm cultivars, thus speeding up the breeding process.

## 2. Results

### 2.1. Salt Stress Treatment and Phenotypic Screening of Elm Cultivars

To evaluate the salt tolerance of elms, one-month-old elm seedlings of 13 cultivars from 13 regions of Shandong, China, exhibiting uniform growth, were randomly selected for control and salt-stress treatment groups. No significant phenotypic changes were observed following 10 days of pretreatment with 200 mM NaCl; however, after 20 days of treatment with 400 mM NaCl, significant phenotypic differences were observed between the different varieties (Figures S1 and S2; Table S1). Under 400 mM NaCl stress, J cultivars displayed the earliest phenotypic response, with leaves curling downwards after 16 days (Figure S2B). By day 18, severe leaf curling was observed in most cultivars, with J cultivars becoming almost completely leafless; in contrast, Y, Q and Z cultivars showed minimal leaf deformation, demonstrating a high level of salt tolerance (Figure S2C). After 20 days, the J cultivars underwent complete defoliation and the JX and L cultivars displayed severe leaf wilting (Figure S2D). Among the salt-tolerant cultivars (Y, Q and Z), Y was the only cultivar to maintain fully expanded leaves throughout the 22-day stress period, showing no visible signs of morphological changes induced by stress (Figure S2D). While cultivars Y, Q and Z demonstrated superior tolerance to salt stress, they exhibited significantly slower growth rates than salt-sensitive cultivars under control conditions during the 22-day observation period (Figure S2E,F).

**Figure 1:**
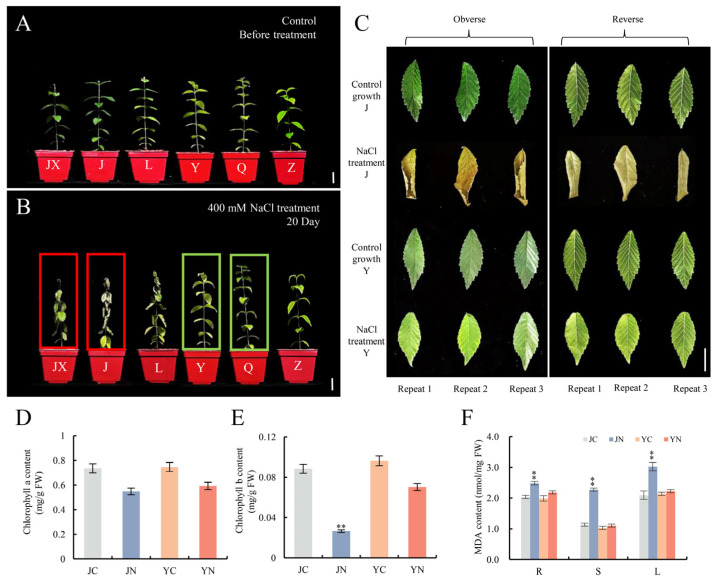
Differential responses of elm cultivars to 400 mM NaCl stress. (**A**,**B**) Phenotypic comparison between control and salt-treated groups (cultivars J, JX, L, Y, Q and Z) at 0 days (**A**) and 20 days (**B**), respectively. Red boxes highlight salt-sensitive cultivars (SS-J and SS-JX) showing severe stress symptoms (leaf wilting, growth inhibition), while green boxes indicate salt-tolerant cultivars (ST-Y and ST-Q) maintaining normal morphology. (**C**) Leaf morphology (the 3rd to 5th fully expanded leaves counted from bottom to top) comparison between SS-J (upper panels) and ST-Y (lower panels) cultivars under control and salt-stressed conditions. (**D**,**E**) Chlorophyll a and b content in leaves after 10 days of treatment. (**F**) MDA accumulation in roots (R), stems (S), and leaves (L) after 10 days of treatment. JC/JN: control/NaCl-treated group of salt-sensitive cultivar SS-J; YC/YN: control/NaCl-treated group of salt-sensitive cultivar ST-Y. Scale bars in (**A**,**B**) = 3 cm; in (**C**) = 1 cm. Data represent mean ± SD; asterisks denote significant differences from control (** *p* < 0.01, Student’s *t*-test).

Following preliminary screening of 13 elm cultivars under salt stress, three salt-tolerant cultivars (Y, Q and Z) and three salt-sensitive cultivars (J, JX and L) were selected for further analysis (Figure 1). After 20 days of 400 mM NaCl treatment, the salt-sensitive cultivars displayed severe symptoms, with J cultivars exhibiting the most severe wilting and leaf yellowing, followed by JX and L cultivars (Figure 1A–C). In contrast, the salt-tolerant cultivars (Y, Q and Z) maintained leaf integrity; Y showed optimal growth, while Q exhibited a moderate level of tolerance (Figure 1A–C). To elucidate potential physiological differences, the salt-tolerant cultivar Y (ST-Y) and the salt-sensitive cultivar J (SS-J) plants were harvested after 10 days of salt treatment to detect chlorophyll and MDA content. As shown in Figure 1, ST-Y maintained stable leaf chlorophyll content and exhibited only a slight increase in MDA levels in the roots, stems and leaves (Figure 1D,E), indicating minimal oxidative damage. This suggested that ST-Y may activate antioxidant defenses via low-level MDA signaling, thereby enhancing stress tolerance. In contrast, SS-J exhibited a significant reduction in chlorophyll b content (70% compared to controls), while chlorophyll a content showed no statistically significant change under salt stress (Figure 1D,E). Additionally, SS-J displayed significantly higher MDA levels across different organs (1.22-fold increase in roots; 2.00-fold increase in stems; 1.44-fold increase in leaves), indicating systemic oxidative damage. We therefore hypothesized that the ST-Y cultivar may be more resistant to stress due to the involvement of the oxidative stress system, whereas the SS-J cultivar may be more sensitive due to the collapse of its oxidative stress system.

### 2.2. ROS Homeostasis in Contrasting Elm Cultivars Under Salt Stress

**Figure 2:**
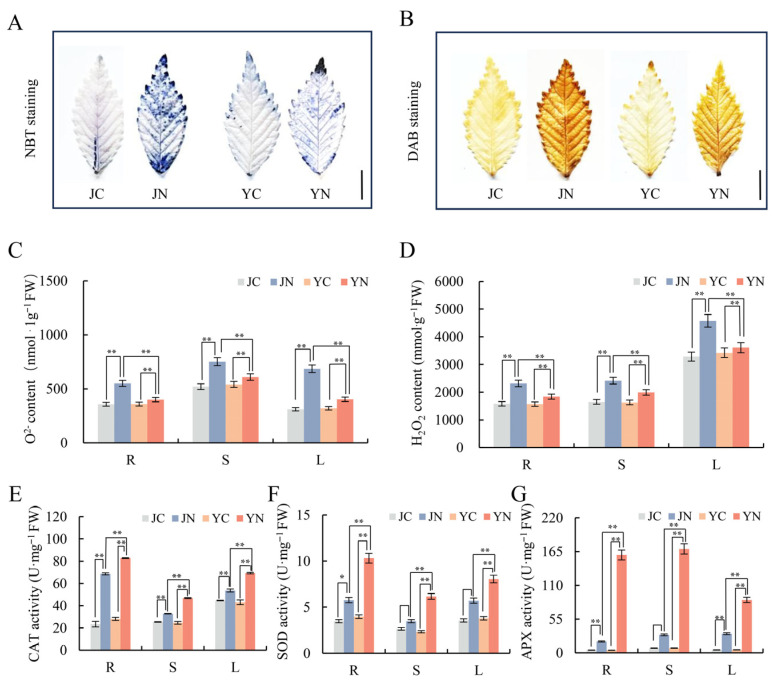
Contrasting ROS regulation and antioxidant defenses in salt-tolerant (ST-Y) versus salt-sensitive (SS-J) elm cultivars under 400 mM NaCl stress. (**A**,**B**) Histochemical detection of O_2_^−^ (NBT staining) and H_2_O_2_ (DAB staining) in leaves of one-month-old seedlings after 10 days of 400 mM NaCl treatment, respectively. Staining reactions were performed at 25 °C (8 h for DAB, 30 min for NBT). (**C**,**D**) Quantitative analysis of O_2_^−^ and H_2_O_2_ accumulation in roots (R), stems (S), and leaves (L), respectively. (**E**–**G**) Activities of antioxidant enzymes (CAT, SOD and APX) in response to salt stress. Scale bar in A = 1 cm. Data represent mean ± SD, and asterisks indicate statistically significant differences between control and treated groups (* *p* < 0.05, ** *p* < 0.01).

Various abiotic stresses universally induce the accumulation of ROS, which subsequently causes oxidative damage that impairs plant growth and development [27,28]. To investigate the spatial accumulation of ROS under salt stress, we analyzed the accumulation of superoxide anion (O_2_^−^) and hydrogen peroxide (H_2_O_2_) in the first fully expanded leaves of ST-Y and SS-J cultivars with NBT (nitroblue tetrazolium) and DAB (3,3′-diaminobenzidine), respectively. And, histochemical analysis revealed that the staining range and signal intensity of O_2_^−^ and H_2_O_2_ in the leaves of SS-J cultivars were significantly larger and stronger than those of ST-Y after salt stress for 10 days (Figure 2A,B), which suggested that ST-Y may maintain the balance of ROS in the leaves through effective antioxidant regulation. Meanwhile, quantitative analysis of O_2_^−^ and H_2_O_2_ accumulation was measured spectrophotometrically across the root, stem, and leaf of both ST-Y and SS-J cultivars at 10 days after salt stress. As shown in Figure 2C,D, the content of O_2_^−^ and H_2_O_2_ in roots, stems, and leaves of salt-treated ST-Y and SS-J cultivars (ST-YN and SS-JN) was significantly higher than that of their respective controls (ST-YC and SS-JC). However, the accumulation of ROS in all organs of SS-J cultivars was significantly higher than that of ST-Y, suggesting that ST-Y cultivars increased salt resistance by reducing the accumulation of ROS. Meanwhile, we analyzed the enzyme activities of ROS scavenging-related enzymes in different organs and showed that CAT, SOD, and APX activities were significantly higher in the SS-J compared to the control but lower than those in the ST-Y after salt-stress treatment (Figure 2E–G). These data demonstrated that ST-Y could activate the antioxidant enzyme system more efficiently under salt stress via timely scavenging of ROS, whereas the antioxidant enzyme system of the SS-J had an insufficient response to control the accumulation of reactive oxygen species to reduce the resistance effectively.

### 2.3. Organ-Specific Transcriptional Adaptation in Response to Salt Stress

To clarify the molecular reasons for the differing salt tolerance levels of SS-J and ST-Y, we performed a detailed assessment through transcriptomes of roots, stems and leaves from one-month-old plants cultivated in soil and exposed to salt stress (400 mM NaCl, 10 days). High-quality transcriptomes were obtained from all 36 samples (Q30 > 95%), yielding 78.46 Gb of clean data. Principal component analysis (PCA) showed clear separation by different cultivars and treatment (Figure S3), confirming data robustness for differential expression analysis.

**Figure 3:**
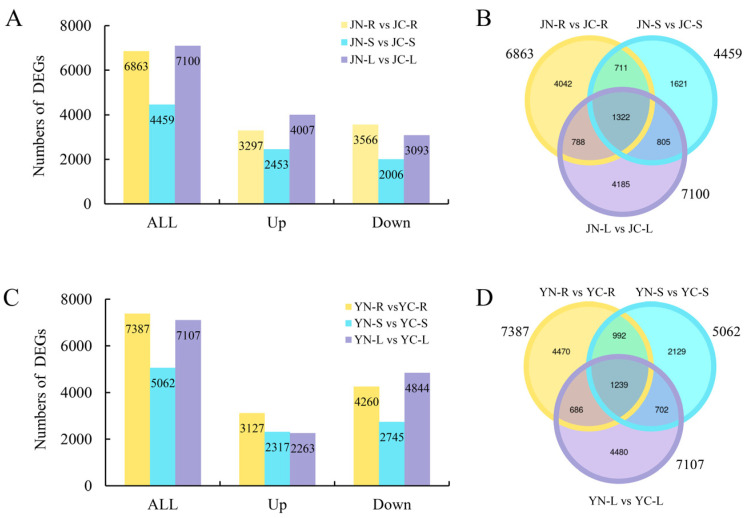
Tissue-specific transcriptional profiling in elm under salt stress. (**A**) SS-J exhibited strong organ-dependent responses, with leaves showing maximal differential expression. (**B**) Venn diagram showing tissue-specific overlaps of DEGs in SS-J after salt treatment. (**C**) ST-Y displayed a more balanced adaptation, with roots mounting the strongest transcriptional response. Roots, R (yellow); stems, S (blue); leaves, L (purple). (**D**) Venn diagram showing tissue-specific overlaps of DEGs in ST-Y after salt treatment. Three independent biological replicates were performed.

The distribution characteristics of DEGs in different organs were quantified by comparing the transcriptome data of root (R), stem (S) and leaf (L) tissues from two elm cultivars in both control and salt-stressed conditions. In detail, the DEGs of SS-J most enriched in the leaf with the highest number of DEGs (JN-L vs. JC-L: 7100), significantly exceeding counts in roots (6863) and stems (4459) (Figure 3A), with only 1322 DEGs shared across all organs (Figure 3B). Notably, a significantly higher number of DEGs were shared by leaves and roots (788), leaves and stems (805) in comparison to roots and stems (711), suggesting that salt sensitivity of SS-J may be closely related to changes in gene expression in leaves. Interestingly, the number of DEGs in ST-Y was more than SS-J after salt-treated, and the DEGs mainly enriched in the roots of the ST-Y (Figure 3C). Additionally, the overlap between roots and stems (992) was more than leaves and stems (686) or leaves and roots (702), reflecting the coordinated regulation of ST-Y at the root level (Figure 3D). Together, ST-Y cultivars optimized the salt tolerance mechanism through a root-dominated transcriptional changes, while SS-J cultivars exacerbated salt sensitivity due to dysregulated leaf gene networks.

### 2.4. Pathway of Salt-Responsive Genes in SS-J and ST-Y via KEGG Enrichment Analysis

To compare the differences in molecular regulatory mechanisms between the ST-Y and the SS-J cultivars under salt stress, we analyzed DEGs in the roots (R), stems (S) and leaves (L). Leaves, as the primary organ responsible for sensing and responding to environmental stresses, directly influence the salinity tolerance of plants [29,30]. Consistent with this, our phenotypic observations after salt stress treatment revealed significant differences: the leaves of the ST-Y cultivars remained green after 20 days of treatment with 400 mM NaCl, whereas the leaves of the SS-J cultivars showed obvious dehydration and browning (Figure 1B). Therefore, analyzing the different pathways through which leaves respond to salt stress provides a deeper understanding of the molecular mechanisms underlying differences in salt tolerance between ST-Y and SS-J cultivars.

**Figure 4:**
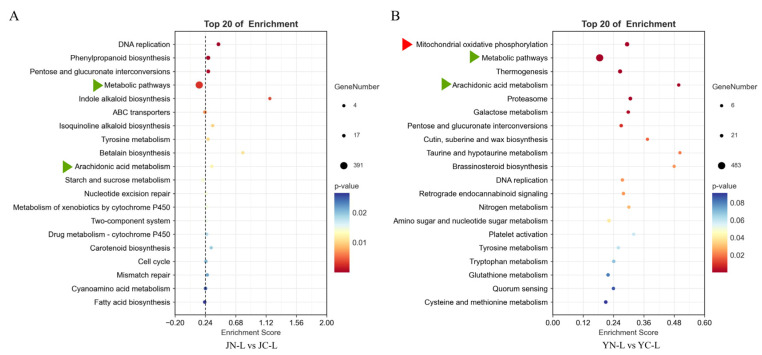
KEGG pathway enrichment analysis of leaf-specific DEGs in elm under salt stress. (**A**) SS-J showed predominant enrichment in metabolic regulation pathways. (**B**) ST-Y exhibited preferential activation of metabolic pathways and mitochondrial oxidative phosphorylation pathways. Red triangles represented specific enrichment pathways in ST-Y; Green triangles indicate pathways co-enriched in both ST-Y and SS-J cultivars.

As shown in Figure 4, transcriptome analysis of the KEGG pathway revealed a unique enrichment of DEGs in the “mitochondrial oxidative phosphorylation (OXPHOS)” pathway of ST-Y leaves after salt stress, suggesting that it may respond to salt stress-induced oxidative stress by increasing the efficiency of ATP synthesis in the electron transport chain. Additionally, both cultivars (ST-Y and SS-J) were highly enriched in “metabolic pathways”, suggesting that elm leaves mainly respond to salt stress by altering some key metabolic pathways. Among metabolic pathways, both cultivars were also enriched in the “arachidonic acid (AA) metabolism”, but the number of DEGs in ST-Y was significantly more than in the SS-J cultivars (Figure 4A,B). Collectively, these findings provide novel insights into the molecular mechanisms underpinning salt tolerance in elm trees.

### 2.5. Coordinated Regulation of Oxidative Phosphorylation and Arachidonic Acid Metabolism

**Figure 5:**
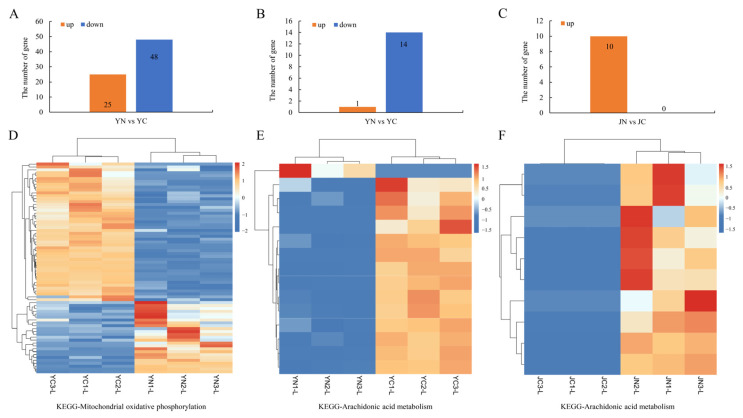
Number and expression patterns of DEGs in the OXPHOS and AA metabolism pathways. (**A**–**C**) The number of up- and down-regulated DEGs in the OXPHOS pathway and AA metabolism pathway in ST-Y and SS-J after salt treatment, respectively. (**D**–**F**) Heatmap of DEGs involved in the OXPHOS and AA metabolism pathways in leaves of ST-Y and SS-J, respectively.

KEGG pathway analysis revealed significant alterations in the “OXPHOS” and “AA metabolism” pathways in the leaves of ST-Y and SS-J cultivars. Analysis of gene expression changes within these pathways showed that 25 genes of the “OXPHOS” pathway were found to be upregulated and 48 of the “OXPHOS” pathway were found to be downregulated in the leaves of ST-Y after salt treatment (Figure 5A), indicating an enhancement of mitochondrial energy metabolism and a synergistic scavenging of ROS in ST-Y. In the “AA metabolism pathway”, 14 down-regulated and one was up-regulated in the leaves of ST-Y after salt treatment (Figure 5B, Table S2), whereas only 10 genes were up-regulated in the leaves of SS-J after salt treatment (Figure 5C; Table S3).

### 2.6. Transcriptomic Validation Through RT-qPCR Analysis

**Figure 6:**
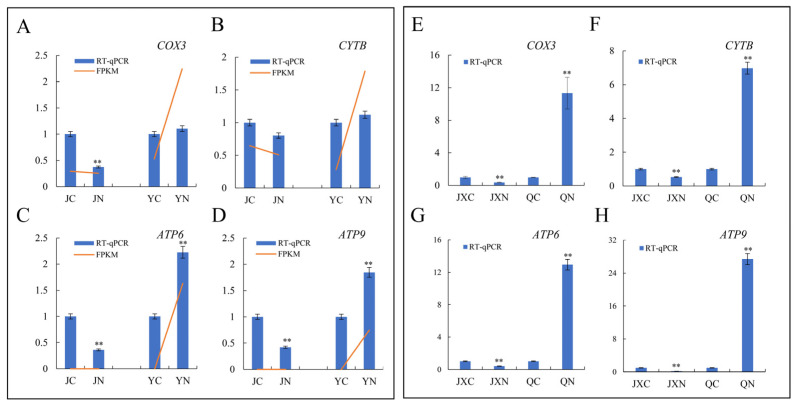
Validation of DEGs using RT-qPCR. (**A**–**D**) RT-qPCR analysis of key genes (*COX3*, *CYTB*, *ATP6* and *ATP9*) in salt-tolerant (YN vs. YC) and salt-sensitive (JN vs. JC) groups. (**E**–**H**) Validation in additional cultivars (QN vs. QC, JXN vs. JXC). Comparisons of RNA-seq data (orange lines) with RT-qPCR (blue bars) of selected genes. Data represent mean ± SD, and asterisks indicate statistically significant differences between control and treated groups (** *p* < 0.01).

To verify the reliability of the RNA-seq data, we used RT-qPCR to quantify the expression of OXPHOS-related genes (such as *COX3*, *CYTB*, *ATP6* and *ATP9*) in the leaves of ST-Y/Q and SS-J/JX cultivars (Figure 6). The results showed that the expression of OXPHOS-related genes in ST-Y plants increased significantly after salt stress. In contrast, gene expression decreased in SS-J after salt stress (Figure 6A–D). Moreover, the expression of OXPHOS-related genes was further validated in ST-Q and SS-JX cultivars after salt treatment, and gene expression in the ST-QN group was increased compared to the ST-QC group, while the expression level in the SS-JXN group was decreased after salt treatment (Figure 6E–H). The RNA-seq data were confirmed by RT-qPCR and further revealed that the OXPHOS pathway genes were differentially regulated by ST-Y/Q and SS-J/JX, providing mechanistic insights into their contrasting salt-stress responses.

## 3. Discussion

Soil salinity is escalating due to ecological degradation and global climate change, posing a severe and growing threat to agricultural productivity and ecosystem stability, which significantly constrains plant growth and development [31]. Therefore, identifying the molecular and physiological mechanisms underlying plant salt tolerance is crucial for developing resilient crops [32]. Elm, as a keystone species, is important for ecological restoration and saline-land afforestation [33]. Previous studies have shown that there are significant differences in salt tolerance among different cultivars of elm trees [5]. Therefore, understanding the molecular mechanisms underlying the differences in salt tolerance among different cultivars of elm trees is crucial for the breeding of salt-tolerant elm trees.

Here, we evaluated salt tolerance of one-month-old seedlings from all 13 locations, which were equally divided into two groups (control group and salt-treated group). Control plants received 50 mL of distilled water every 4 days, while salt-treated plants were gradually acclimated to saline conditions: initially irrigated with 50 mL of 200 mM NaCl solution for the first 10 days, followed by an increase to 400 mM NaCl maintained at the same irrigation interval until the end of the experiment (Figure S1). Phenotypic observations were performed at 16, 18 and 22 days after treatment; based on initial observations, three salt-tolerant and three salt-sensitive cultivars were selected for further salt stress treatment with continued phenotypic monitoring. After 20 days, two contrasting cultivars were identified: the salt-tolerant ST-Y/Q and the salt-sensitive SS-J/JX (Figure S2A–D; Figure 1B). It should be noted that the 400 mM NaCl concentration used for salt stress treatment was determined based on our preliminary experiment: in the preliminary test, 13 elm cultivars were treated with 200 mM and 400 mM NaCl, respectively; after 20 days of 200 mM NaCl treatment, all cultivars exhibited strong salt tolerance with no obvious stress symptoms, making it impossible to distinguish salt-tolerant from salt-sensitive genotypes, whereas 400 mM NaCl treatment for 20 days resulted in significant phenotypic variations among different elm cultivars, enabling clear identification of ST-Y/Q and SS-J/JX. Chloroplasts are vital for photosynthesis and other biosynthetic processes in plant cells [34], and their integrity and function are greatly affected by salinity stress, causing impaired cellular function [35]. It is worth noting that 400 mM NaCl has been used as a high-salt treatment concentration for woody plants in previous studies, such as a recent study on *Populus talassica × Populus euphratica*, which similarly employed this concentration to elucidate the salt tolerance mechanisms in poplars [36]. However, the applicability of these findings under moderate salt stress or field conditions still requires further validation. In this study, our results showed that the ST-Y effectively preserved leaf integrity and chlorophyll levels under salt stress, which was associated with reduced ROS accumulation and decreased oxidative damage (Figure 1D–F). In contrast, the SS-J showed pronounced leaf chlorosis and curling after 20 days of salt treatment (Figure 1B). This pattern of differential tolerance was consistent with the previous findings, such as the enhanced salt resistance observed in tetraploid *Robinia pseudoacacia* compared to diploid plants due to the ability to maintain chlorophyll stability and retain chloroplast structural integrity in tetraploid *Robinia pseudoacacia* [37].

Salt stress always induces the accumulation of ROS in plant cells, and low levels of ROS can activate signaling pathways; excessive accumulation can cause oxidative damage to the cellular membrane [38]. And, antioxidant enzymes (such as SOD, POD, CAT and APX) were also known to increase for removing ROS from plant cells [39]. Previous studies have shown that salt-tolerant rice (Oryza sativa) cultivars have higher levels of SOD and POD activities compared to salt-sensitive cultivars [40,41]. Similar results were obtained in this study: salt stress generally increased the activities of antioxidant enzymes in both cultivars (Figure 2E–G). However, ST-Y exhibited stronger antioxidant activity under salt stress, resulting in lower O_2_^−^ and H_2_O_2_ content than the SS-J (Figure 2C,D). In addition, the accumulation of excess ROS led to membrane lipid peroxidation, with MDA being a key indicator of this process [42]. Previous studies have shown that MDA levels increase in salt-sensitive IR29 rice seedlings under prolonged salt stress [43]. Similarly, the content of MDA in SS-J was significantly increased after salt treatment, suggesting that SS-J was a salt-sensitive elm cultivar (Figure 1F).

To investigate organ-specific molecular responses to salt stress in ST-Y and SS-J cultivars, we performed transcriptome analyses. Similar research approaches have successfully resolved salt stress response mechanisms in other species. For example, comparing the transcriptome data of different salt-tolerant maize (Zea mays) cultivars revealed that salt stress significantly alters the expression profiles of transcription factors. The identified salt-tolerance-related genes can be used as targets in breeding programs [44]. In rice (*Oryza sativa*), comparing the transcriptomes of the salt-sensitive variety Giza 177 with the salt-tolerant variety Giza 178 showed that differentially expressed genes (DEGs) were mainly enriched in the cell wall modification pathway [45]. In the present study, analyzing the number of DEGs in ST-Y and SS-J revealed that SS-J had a significantly higher number of DEGs in leaves (Figure 3A,B), whereas ST-Y exhibited a significantly higher number of DEGs in roots (Figure 3C,D). Although ST-Y exhibited a root-dominated transcriptional response, we prioritized leaf analysis because phenotypic divergence was most distinct there (Figure 1), OXPHOS enrichment was leaf-specific (Figure 4), and leaves are the primary site of photosynthesis and ROS generation. We acknowledge root-specific mechanisms remain critical for future investigation. Subsequently, KEGG pathway analysis indicated that ST-Y displayed a distinct metabolic response to salt stress in leaves, particularly within the OXPHOS pathway (Figure 4A,B). This finding aligns with the previous reports of OXPHOS involvement in saline and heat stress responses in tomatoes [46]. Therefore, we hypothesize that ST-Y enhanced cell membrane stability may be achieved through the regulation of phospholipase activity or oxidative lipid synthesis, potentially contributing to the maintenance of leaf morphological integrity observed (Figure 1C).

Further analysis revealed that DEGs associated with the OXPHOS pathway were significantly upregulated in ST-Y/Q, including encoding mitochondrial complex IV (COX3), ATP synthase subunits (ATP6/ATP9), and cytochrome b (CYTB) (Figure 6A–H). Conversely, these OXPHOS-related DEGs were significantly downregulated in SS-J/JX (Figure 6A–H). It should be noted that the RNA-seq data showed that OXPHOS-related genes were both up- and down-regulated, and simply reporting transcript changes is insufficient to prove that OXPHOS pathways are crucial for salt tolerance. To substantiate this claim, functional evidence linking OXPHOS status to salt-stress physiology is required. Electron leakage from the mitochondrial electron transport chain (ETC) during OXPHOS is the main source of superoxide production; tightly coupled OXPHOS reduces ETC electron leakage and mitochondrial ROS generation [47,48]. The lower ROS accumulation in ST-Y directly reflects optimized ETC function, and stable and efficient OXPHOS under stress prevents ROS overproduction and membrane lipid peroxidation caused by mitochondrial dysfunction. Thus, the reduced ROS and MDA levels observed in ST-Y can indirectly indicate the functional status of the OXPHOS pathway (Figure 2). Therefore, we speculated that the enhanced ATP synthesis efficiency would be for powering processes like the Na^+^/H^+^ antiporter and other energy-consuming activities, while concurrently reducing electron leakage and subsequent O_2_^−^ generation. This synergistic optimization of energy production and ROS regulation is consistent with findings in saline-tolerant flora [49].

In addition to energy metabolism, our KEGG enrichment analysis identified the AA metabolism pathway as significantly enriched in response to salt stress (Figure 4, Tables S2 and S3). Although the “AA metabolic” pathway was enriched in both cultivars following treatment, the level of enrichment was markedly higher in ST-Y than in SS-J. While plants generally possess low endogenous levels of AA compared to animals, and many plant AA metabolism genes involve lipid oxygenation pathways, AA acts as a conserved signaling molecule in plants [25]. It plays a vital role in regulating stress signaling networks by balancing the JA and SA pathways. Given the significant enrichment in the salt-tolerant cultivar, we hypothesize that the AA metabolism pathway in elm is a critical component of the salt stress response. The differential enrichment between ST-Y and SS-J suggests that the modulation of AA-derived signaling may contribute to the superior stress adaptability observed in salt-tolerant elm cultivars. Collectively, these findings highlight that salt tolerance in elm is governed by a complex interplay of maintained energy metabolism (OXPHOS) and specialized lipid signaling (AA metabolism).

## 4. Materials and Methods

### 4.1. Plant Materials and Salt Treatments

One-month-old elm seedlings were collected from 13 locations representing Shandong Province’s major bioclimatic regions, and the collection details for all 13 elm seedlings are shown in Supplementary Table S1. For each of the 13 cultivars, three biological replicates were established, with 10 seedlings per replicate, resulting in a total of 30 seedlings per cultivar used in the experiment. The seedlings were planted in 10 × 10 × 5 cm pots containing a nutrient substrate cultivated under controlled greenhouse conditions (day/night temperature of 26/22 °C, 16 h photoperiod, and relative humidity of 40–50%).

To facilitate gradual acclimation to saline conditions, salt-treated plants were initially irrigated with 50 mL of 200 mM NaCl solution for the first 10 days. Subsequently, the concentration was increased to 400 mM NaCl and maintained at equivalent irrigation intervals until the end of the experiment. Control plants received 50 mL of distilled water every 4 days. Phenotypic observations were performed at 16, 18 and 22 days after treatment. Based on initial observations, three salt-tolerant and three salt-sensitive cultivars were selected for subsequent analysis. The above six cultivars were subjected to further salt stress treatment, with continued phenotypic observation. After 20 days, two contrasting cultivars were identified: the ST-Y/Q and the SS-JX/J. We quantified morphological alterations and physiological traits in the leaves (the 3rd to 5th fully expanded leaves counted from bottom to top) of representative ST-Y and SS-J. For the transcriptomic analysis, root, stem and leaf tissues were collected from both control and treated seedlings at 10 days after treatment, and these tissues were then immediately flash-frozen in liquid nitrogen and stored at −80 °C until RNA extraction.

### 4.2. Chlorophyll Content and Physiological Stress Response Indicator Analysis

The selection of roots, stems (internodes 3 to 8 of the culm) and leaves (5th to 7th fully expanded leaves) from ST-Y and SS-J elms was for the measurement of malondialdehyde (MDA) and chlorophyll content under both control and salt-treated conditions. For chlorophyll analysis, 0.5 g of fresh weight leaf cultivars were homogenized in 95% ethanol, transferred to a 5 mL centrifuge tube and centrifuged. For chlorophyll quantification, the supernatant was diluted to a final volume of 8 mL with extraction buffer. Chlorophyll a and b concentrations were determined spectrophotometrically at 663 nm and 645 nm, respectively. The MDA content of the roots, stems and leaves of both ST-Y and SS-J plants was quantified using a thiobarbituric acid (TBA) assay kit, in accordance with the manufacturer’s instructions (Nanjing Jiancheng Bioengineering Institute, Nanjing, China) [50]. Similarly, the activities of SOD, CAT and APX were determined using the relevant commercial kits from the same manufacturer.

### 4.3. Histochemical Staining and Quantification of ROS

The histochemical localization of H_2_O_2_ and O_2_^−^ was examined in the first fully expanded leaves of one-month-old elm seedlings, after a 10-day salt treatment period. H_2_O_2_ was detected using a 3,3′- DAB stain (Coolaber, item number SL1805), while O_2_^−^ was detected using an NBT stain (Coolaber, item number SL18061). The procedure was as follows: Histochemical staining was performed as follows: (1) Leaf cultivars were vacuum-infiltrated (15 min) with either DAB (1 mg/mL) or NBT (1 mg/mL) staining solutions; (2) Staining reactions were completed at 25 °C (8 h for DAB, 30 min for NBT); (3) Chlorophyll was removed by incubating cultivars in 95% ethanol at 80 °C until complete decolorization occurred.

To quantify the accumulation of O_2_^−^ and H_2_O_2_ in different organs in ST-Y and SS-J elm cultivars, we collected samples of mature leaves (5th to 7th fully expanded leaves), stems (internodes 3 to 8 of the culm), and roots from one-month-old plants after 10 days of salt treatment. The H_2_O_2_ content was quantified using the titanium sulfate colorimetric method kit, while O_2_ˉ levels were determined using a commercial assay kit (Inhibition and Production of superoxide anion assay kit, A052-1-1), both of which were obtained from the Nanjing Jiancheng Bioengineering Institute (Nanjing, China).

### 4.4. RNA Isolation and Real-Time Quantitative PCR (RT-qPCR) Analysis

Total RNA was isolated from 36 samples, including roots, stems and leaves from both the ST-Y and SS-J cultivars under both control and salt-treated conditions (*n* = 3 biological replicates per cultivar), using the FastPure Universal Plant Total RNA Extraction Kit (Vazyme, Nanjing, China). RNA integrity was verified by agarose gel electrophoresis and Nanodrop spectrophotometry before library preparation and sequencing (RNA-Seq), which was performed by Shanghai Ouyi Biomedical Technology Co., Ltd. (Shanghai, China). To validate the genes, 1 μg of the total RNA was reverse-transcribed using HiScript III All-in-One RT SuperMix (Vazyme). RT-qPCR was performed in quadruplicate technical replicates using SYBR Premix Ex Taq (Takara, Shiga, Japan) on a Bio-Rad CFX Connect system (Hercules, CA, USA). Following the manufacturer’s instructions, the thermal cycling conditions were as follows: initial denaturation at 95 °C for 30 s, followed by 40 cycles of 95 °C for 5 s and 60 °C for 30 s. A melt curve analysis was performed by heating the amplicon from 65 °C to 95 °C to confirm primer specificity. 18S rRNA was used as the internal control and gene-specific primers were designed using Primer Premier 3.0 (https://www.primer3plus.com/, (accessed on 15 March 2025)) (Table S4) [5]. Relative expression was calculated via the delta-delta Ct (2^−ΔΔCt^) method.

### 4.5. RNA-Seq and Differentially Expressed Genes (DEGs) Analysis

RNA-seq libraries were constructed and sequenced on the Illumina NovaSeq 6000 platform at Novogene in Beijing, China. Their integrity was then examined using an Agilent 2100 Bioanalyzer (Agilent Technologies, Santa Clara, CA, USA). Raw reads were filtered to remove aptamers and low-quality bases (Q < 5). The transcriptome was then reassembled using Trinity, generating 91,204 single genes (N50 = 1688 bp). Gene expression was quantified as FPKM using RSEM after comparing clean reads to single genes (Bowtie2). DEGs were identified using edgeR to analyze salt sensitivity and salt tolerance in the J and Y cultivars. Screening thresholds were set at |log_2_ FC| > 2 and a q value < 0.05. This was followed by functional enrichment analysis (GO/KEGG) and visualization (PCA and heatmap).

### 4.6. Statistical Analysis

All experiments were performed with three independent biological replicates. Data are presented as mean ± standard deviation (SD). Statistical significance was determined by Student’s *t*-tests using Microsoft Excel (v.2019), with significance levels set at * *p* < 0.05 and ** *p* < 0.01.

## 5. Conclusions

This study selected two salt-tolerant (ST-Y/Q) and two salt-sensitive (SS-J/JX) elm cultivars from 13 regions in Shandong Province by conducting a phenotypic analysis after exposing the plants to salt stress. Phenotypic and physiological indicator analyses were then conducted on one-month-old ST-Y and SS-J elm cultivars to reveal the effects of salt stress. Subsequently, transcriptomic profiling revealed organ-specific responses in ST-Y and SS-J elm trees during salt stress: main DEGs of ST-Y were concentrated in the roots, while SS-Js were primarily enriched in the leaves. KEGG pathway analysis of the DEGs showed that those in the leaves of both cultivars were enriched in “metabolic pathways” following salt treatment. Among metabolic pathways, the “AA metabolism pathway” exhibited significantly higher enrichment in ST-Y than in SS-J. Furthermore, DEGs in ST-Y were specifically enriched in the “OXPHOS” pathway. RT-qPCR analysis revealed that OXPHOS-related genes were upregulated in ST-Y/Q, but downregulated in SS-J/JX. This study indicated that salt-tolerant elm cultivars (ST-Y/Q) respond to salt stress through the synergistic regulation of the antioxidant system and the “OXPHOS” pathway. These results provide valuable genetic resources and a theoretical foundation for developing new salt-tolerant elm cultivars through targeted molecular breeding techniques.

## Data Availability

The sequenced clean reads generated in this study have been deposited in the Chinese Academy of Sciences (GSA: CRA023620), which is publicly accessible at https://bigd.big.ac.cn/gsa/browse/CRA023620 (accessed on 21 March 2025).

## Supplementary Materials

The following supporting information can be downloaded at: https://www.mdpi.com/article/10.3390/plants15081164/s1, Table S1: Samples from 13 districts in Shandong Province, along with information on their sampling points. Table S2: Enrichment analysis of the KEGG pathway in DEGs of JN vs. JC leaves. Table S3: Enrichment analysis of the KEGG pathway in DEGs of YN vs. YC leaves. Table S4: Primer sequences were used in this study. Figure S1: Phenotypic responses of 13 elm accessions under 200 mM NaCl treatment. Figure S2: Phenotypic responses of 13 elm cultivars under salt stress. Figure S3: Principal component analysis (PCA) of transcriptomic data from elm tissues.

## References

[1] Liang Y., Liu H., Fu Y., Li P., Li S., Gao Y. (2023). Regulatory effects of silicon nanoparticles on the growth and photosynthesis of cotton seedlings under salt and low-temperature dual stress. BMC Plant Biol..

[2] Bao Y., Ma B., McLaughlin N.B., Niu Y., Wang D., Liu H., Li M., Sun Z. (2024). The impact of salinization on soil bacterial diversity, yield and quality of Glycyrrhiza uralensis Fisch. Front. Microbiol..

[3] Wang Z., Tan W., Yang D., Zhang K., Zhao L., Xie Z., Xu T., Zhao Y., Wang X., Pan X. (2021). Mitigation of soil salinization and alkalization by bacterium-induced inhibition of evaporation and salt crystallization. Sci. Total Environ..

[4] Wang L., Zhao Z.Y., Zhang K., Tian C.Y. (2013). Reclamation and utilization of saline soils in arid northwestern China: A promising halophyte drip-irrigation system. Environ. Sci. Technol..

[5] Chen P., Liu P., Zhang Q., Bu C., Lu C., Srivastava S., Zhang D., Song Y. (2021). Gene Coexpression Network Analysis Indicates that Hub Genes Related to Photosynthesis and Starch Synthesis Modulate Salt Stress Tolerance in *Ulmus pumila*. Int. J. Mol. Sci..

[6] Yu Z., Song J., Xu L., Zhang H. (2024). Evaluating the Combustion Performance of the Usual Timbers in Furniture Using a Grey Correlation Method Based on Thermolysis, Ignition, and Flame Spread. Fire.

[7] Batsaikhan G.-E., Suren M., Enkhbayar B., Dugarjav D. (2020). Growth and biomass of siberian elm seedlings (*Ulmus pumila* L.) grown in tree nursery. Mong. J. Agric. Sci..

[8] Mu D., Zwiazek J.J., Li Z., Zhang W. (2016). Genotypic variation in salt tolerance of *Ulmus pumila* plants obtained by shoot micropropagation. Acta Physiol. Plant..

[9] Chen C., Chu Y., Huang Q., Zhang W., Ding C., Zhang J., Li B., Zhang T., Li Z., Su X. (2021). Morphological, physiological, and transcriptional responses to low nitrogen stress in Populus deltoides Marsh. clones with contrasting nitrogen use efficiency. BMC Genom..

[10] Niramaya S.M., Ganesh C.N., Nilima S.R., Suprasanna P., Tukaram D.N. (2016). Plant Salt Stress: Adaptive Responses, Tolerance Mechanism and Bioengineering for Salt Tolerance. Bot. Rev..

[11] Zhao S., Zhang Q., Liu M., Zhou H., Ma C., Wang P. (2021). Regulation of Plant Responses to Salt Stress. Int. J. Mol. Sci..

[12] Yalcinkaya T., Uzilday B., Ozgur R., Turkan I., Mano J.i. (2019). Lipid peroxidation-derived reactive carbonyl species (RCS): Their interaction with ROS and cellular redox during environmental stresses. Environ. Exp. Bot..

[13] Yuqi Z., Shuhao L., Shengxiang R., Yang X., Maomao H., Mingxuan H., Fenglin Z. (2024). Genome-Wide Identification and Characterization of the Superoxide Dismutase (SOD) Gene Family in Pakchoi and the Role of the *BchFSD2* Gene in the Salt Stress Toleran. Agronomy.

[14] Li J., Yang Y., Wang F., Ma Q., Jia H. (2025). Magnesium-dependent phosphatase 1 (MDP1) interacts with WRKY 53 and protein phosphatase 2C 80 (PP2C80) to improve salt stress tolerance by scavenging reactive oxygen species in Salix psammophila. Int. J. Biol. Macromol..

[15] Qiang Q., Zhang Z., Li X., Li C., Mao M., Ding X., Zhang J., Li S., Lai Z., Yang J. (2025). The amino acid permease SlAAP6 contributes to tomato growth and salt tolerance by mediating branched-chain amino acid transport. Hortic. Res..

[16] Song Q., He F., Kong L., Yang J., Wang X., Zhao Z., Zhang Y., Xu C., Fan C., Luo K. (2024). The IAA17.1/HSFA5a module enhances salt tolerance in Populus tomentosa by regulating flavonol biosynthesis and ROS levels in lateral roots. New Phytol..

[17] Hao L., Qiling S., Xing L., Peipei G., Daling F., Xiaomeng Z., Yin L., Jingsen Y., Shuxing S., Jianjun Z. (2024). Synergistic effects of carbon cycle metabolism and photosynthesis in Chinese cabbage under salt stress. Hortic. Plant J..

[18] Nath S. (2016). The thermodynamic efficiency of ATP synthesis in oxidative phosphorylation. Biophys. Chem..

[19] Chidozie N.O., Shon A.K., Andrew P.W. (2023). Mitochondrial complex I ROS production and redox signaling in hypoxia. Redox Biol..

[20] Schofield J.H., Schafer Z.T. (2021). Mitochondrial Reactive Oxygen Species and Mitophagy: A Complex and Nuanced Relationship. Antioxid. Redox Signal..

[21] Hashemipetroudi S.H., Ahmadian G., Fatemi F., Nematzadeh G., Yamchi A., Kuhlmann M. (2022). Ion content, antioxidant enzyme activity and transcriptional response under salt stress and recovery condition in the halophyte grass Aeluropus littoralis. BMC Res. Notes.

[22] Tao L.I., Run-Jin L.I.U., Xin-Hua H.E., Bao-Shan W. (2012). Enhancement of Superoxide Dismutase and Catalase Activities and Salt Tolerance of Euhalophyte *Suaeda salsa* L. by Mycorrhizal Fungus Glomus mosseae. Pedosphere.

[23] Zhang Q.-F., Li Y.-Y., Pang C.-H., Lu C.-M., Wang B.-S. (2005). NaCl enhances thylakoid-bound SOD activity in the leaves of C3 halophyte *Suaeda salsa* L.. Plant Sci..

[24] Averill-Bates D. (2024). Reactive oxygen species and cell signaling. Review. Biochim. Biophys. Acta (BBA)-Mol. Cell Res..

[25] Savchenko T., Walley J.W., Chehab E.W., Xiao Y., Kaspi R., Pye M.F., Mohamed M.E., Lazarus C.M., Bostock R.M., Dehesh K. (2010). Arachidonic acid: An evolutionarily conserved signaling molecule modulates plant stress signaling networks. Plant Cell.

[26] Lewis D.C., Stevens D.M., Little H., Coaker G.L., Bostock R.M. (2023). Overlapping Local and Systemic Defense Induced by an Oomycete Fatty Acid MAMP and Brown Seaweed Extract in Tomato. Mol. Plant-Microbe Interact..

[27] Wang P., Liu W.C., Han C., Wang S., Bai M.Y., Song C.P. (2024). Reactive oxygen species: Multidimensional regulators of plant adaptation to abiotic stress and development. J. Integr. Plant Biol..

[28] You J., Chan Z. (2015). ROS Regulation During Abiotic Stress Responses in Crop Plants. Front. Plant Sci..

[29] Wei S.-S., Wang X.-Y., Liu P., Zhang J.-W., Zhao B., Dong S.-T. (2016). Comparative proteomic analysis provides new insights into ear leaf senescence of summer maize (*Zea mays* L.) under field condition. J. Integr. Agric..

[30] Geldhof B., Pattyn J., Eyland D., Carpentier S., Van de Poel B. (2021). A digital sensor to measure real-time leaf movements and detect abiotic stress in plants. Plant Physiol..

[31] Han Z., Liu H., Zhao X., Liu S., Zhang J., Guo S., Wang B., Zhao L., Jin Y., Guo Y. (2024). Functional characterization of maize phytochrome-interacting factor 3 (ZmPIF3) in enhancing salt tolerance in arabidopsis. Sci. Rep..

[32] Zheng Y., Zong J., Liu J., Wang R., Chen J., Guo H., Kong W., Liu J., Chen Y. (2022). Mining for salt-tolerant genes from halophyte Zoysia matrella using FOX system and functional analysis of ZmGnTL. Front. Plant Sci..

[33] Akbari Oghaz N., Rahnama K., Vatandoost H., Afshari A., White J.F., Hyde K.D., Yazdanian M., Salari E., Hatamzadeh S., Taheri A. (2025). Entomopathogenic fungi as guardians of elm trees: A review of dual-action biocontrol agents targeting *Scolytus* spp. and their associated Ophiostoma species. J. Invertebr. Pathol..

[34] Wang L., Liang W., Xing J., Tan F., Chen Y., Huang L., Cheng C.L., Chen W. (2013). Dynamics of chloroplast proteome in salt-stressed mangrove *Kandelia candel* (L.) Druce. J. Proteome Res..

[35] Hameed A., Ahmed M.Z., Hussain T., Aziz I., Ahmad N., Gul B., Nielsen B.L. (2021). Effects of Salinity Stress on Chloroplast Structure and Function. Cells.

[36] Liu Y., Su M., Zhao X., Liu M., Wu J., Wu X., Lu Z., Han Z. (2025). Combined transcriptomic and metabolomic analysis revealed the salt tolerance mechanism of Populus talassica × Populus euphratica. BMC Plant Biol..

[37] Meng F., Luo Q., Wang Q., Zhang X., Qi Z., Xu F., Lei X., Cao Y., Chow W.S., Sun G. (2016). Physiological and proteomic responses to salt stress in chloroplasts of diploid and tetraploid black locust (*Robinia pseudoacacia* L.). Sci. Rep..

[38] Chen Q., Yang G. (2020). Signal Function Studies of ROS, Especially RBOH-Dependent ROS, in Plant Growth, Development and Environmental Stress. J. Plant Growth Regul..

[39] Kesawat M.S., Satheesh N., Kherawat B.S., Kumar A., Kim H.U., Chung S.M., Kumar M. (2023). Regulation of Reactive Oxygen Species during Salt Stress in Plants and Their Crosstalk with Other Signaling Molecules-Current Perspectives and Future Directions. Plants.

[40] Kordrostami M., Rabiei B., Hassani Kumleh H. (2017). Biochemical, physiological and molecular evaluation of rice cultivars differing in salt tolerance at the seedling stage. Physiol. Mol. Biol. Plants Int. J. Funct. Plant Biol..

[41] Duong C.Q., Bui A.L., Le T.H., Tran T.T., Trinh N.N. (2023). Differential biochemical and metabolic responses of contrast rice cultivars (*Oryza sativa* L.) under salt stress. Acta Agrobot..

[42] Del Rio D., Stewart A.J., Pellegrini N. (2005). A review of recent studies on malondialdehyde as toxic molecule and biological marker of oxidative stress. Nutr. Metab. Cardiovasc. Dis..

[43] Moradi F., Ismail A.M. (2007). Responses of photosynthesis, chlorophyll fluorescence and ROS-scavenging systems to salt stress during seedling and reproductive stages in rice. Ann. Bot..

[44] Du X., Wang G., Ji J., Shi L., Guan C., Jin C. (2016). Comparative transcriptome analysis of transcription factors in different maize varieties under salt stress conditions. Plant Growth Regul..

[45] Shehab M., Iovene M., Ciancio A., Colagiero M., Finetti-Sialer M. (2022). Transcriptome Analysis Provides Novel Insights into Salt Stress Response in Two Egyptian Rice Varieties with Different Tolerance Levels. Rice Sci..

[46] Li Y., Jiang F., Niu L., Wang G., Yin J., Song X., Ottosen C.O., Rosenqvist E., Mittler R., Wu Z. (2024). Synergistic regulation at physiological, transcriptional and metabolic levels in tomato plants subjected to a combination of salt and heat stress. Plant J. Cell Mol. Biol..

[47] Zhou H., Shi H., Yang Y., Feng X., Chen X., Xiao F., Lin H., Guo Y. (2024). Insights into plant salt stress signaling and tolerance. J. Genet. Genom..

[48] Wu J., Wang X., Xu J., Li T., Shan G., Zhang L., Yan T., Song X., Sun Y., Guo H. (2026). Overexpression of soybean flavonoid 3′-hydroxylase enhances plant salt tolerance by promoting ascorbic acid biosynthesis. J. Adv. Res..

[49] Nianwei Q., Min C., Jianrong G., Huayin B., Xiuling M., Baoshan W. (2007). Coordinate up-regulation of V-H+-ATPase and vacuolar Na+/H+ antiporter as a response to NaCl treatment in a C3 halophyte *Suaeda salsa*. Plant Sci..

[50] Li J., Xu L., Xuan P., Tian Z., Liu R. (2024). Thiourea and arginine synergistically preserve redox homeostasis and ionic balance for alleviating salinity stress in wheat. Sci. Rep..

