# Supplementary material for: Transcriptome Analysis Unveils the Crucial Role of Mitochondrial Oxidative Phosphorylation Pathways in Ulmus pumila in Response to Salt Stress
Source: Plants (Basel). 2026 Apr 9;15(8):1164. doi: 10.3390/plants15081164 (PMC13119173; doi:10.3390/plants15081164)
Supplement: Supplementary file 1 [file plants-15-01164-s001.zip › Supplementary Figures.pdf]

**Supplementary Figures**

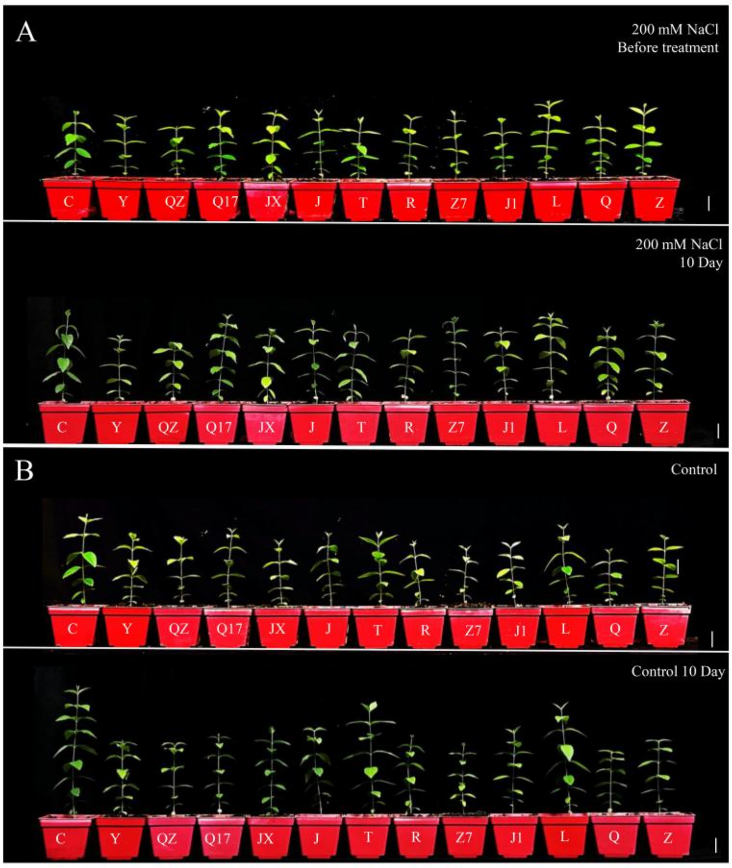


**Figure S1.** Phenotypic responses of 13 elm accessions under 200 mM NaCl treatment. (A) Phenotypes of 13 elm accessions treated with 200 mM NaCl at 0 day (before treatment) and 10 days after treatment. (B) Phenotypes of 13 elm accessions grown under normal conditions (control) at 0 day and 10 days after treatment. Scale bar = 3 cm.

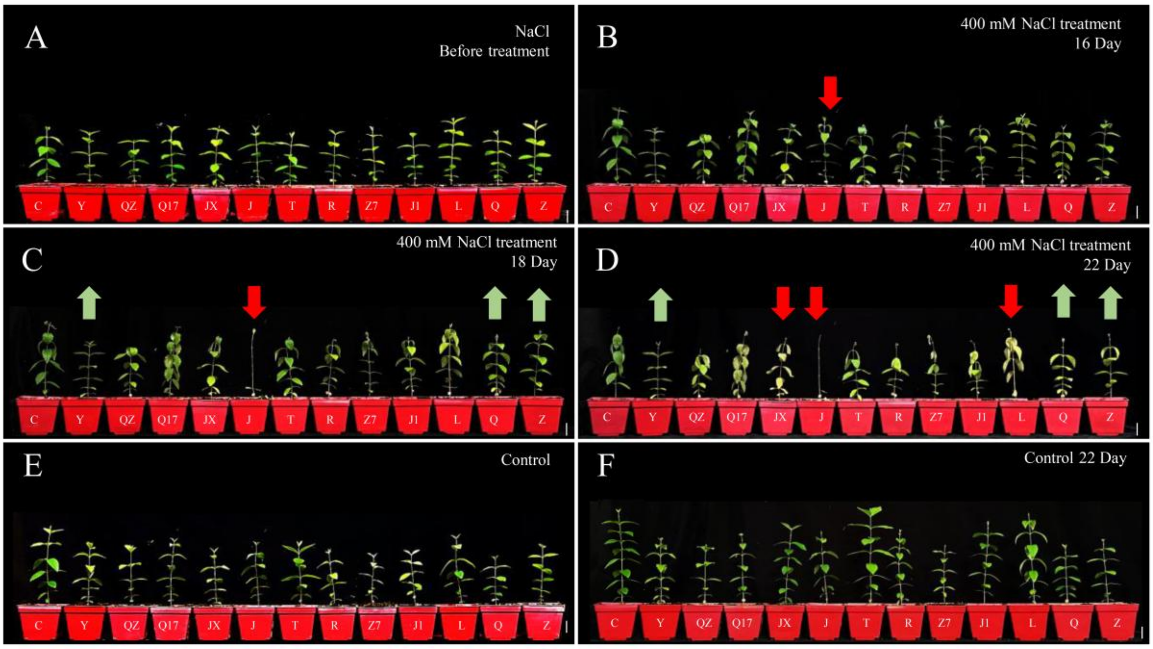

**Figure S2.** Phenotypic responses of 13 elm cultivars under salt stress. **(A)** Initial morphology before salt treatment. (B) Early stress response at 16 days (d), and salt-sensitive cultivars (J, red arrow) exhibit initial leaf curling. (C) Intermediate stress at 18 d, and severe leaf curling and partial defoliation in salt-sensitive cultivar J (red arrow), while salt-tolerant cultivars Y, Q, Z maintain leaf integrity (green arrows). (D) Terminal stress at 22 days, complete defoliation occurred in salt-sensitive cultivars (JX, J, and L red arrow), whereas salt-tolerant cultivars (Y, Q and Z, green arrow) retained leaf integrity. (E) and (F) Photos of the control group, taken before treatment (E) and after 22 days (F) of growth in a normal environment, respectively. Scale bar = 3 cm.

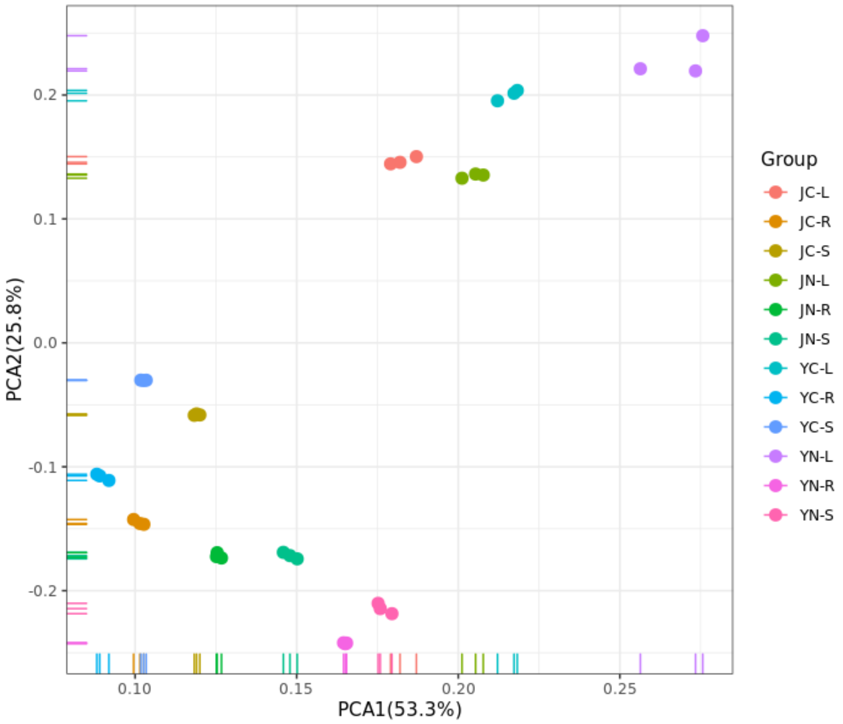


**Figure S3.** Principal component analysis (PCA) of transcriptomic data from elm tissues. JC represents SS-J from the control group, JN represents SS-J from the salt-treated group, YC represents ST-Y from the control group, YN represents ST-Y from the salt-treated group; R represents roots, S represents stems, L represents leaves.
